# NGlyAlign: an automated library building tool to align highly divergent HIV envelope sequences

**DOI:** 10.1186/s12859-020-03901-y

**Published:** 2021-02-08

**Authors:** Elma H. Akand, John M. Murray

**Affiliations:** School of Mathematics and Statistics, UNSW, Sydney, NSW Australia

**Keywords:** HIV, Sequence alignment, Glycosylation, Anchored alignment

## Abstract

**Background:**

The high variability in envelope regions of some viruses such as HIV allow the virus to establish infection and to escape subsequent immune surveillance. This variability, as well as increasing incorporation of N-linked glycosylation sites, is fundamental to this evasion. It also creates difficulties for multiple sequence alignment methods (MSA) that provide the first step in their analysis. Existing MSA tools often fail to properly align highly variable HIV envelope sequences requiring extensive manual editing that is impractical with even a moderate number of these variable sequences.

**Results:**

We developed an automated library building tool NGlyAlign, that organizes similar N-linked glycosylation sites as block constraints and statistically conserved global sites as single site constraints to automatically enforce partial columns in consistency-based MSA methods such as Dialign. This combined method accurately aligns variable HIV-1 envelope sequences. We tested the method on two datasets: a set of 156 founder and chronic gp160 HIV-1 subtype B sequences as well as a set of reference sequences of gp120 in the highly variable region 1. On measures such as entropy scores, sum of pair scores, column score, and similarity heat maps, NGlyAlign+Dialign proved superior against methods such as T-Coffee, ClustalOmega, ClustalW, Praline, HIValign and Muscle. The method is scalable to large sequence sets producing accurate alignments without requiring manual editing. As well as this application to HIV, our method can be used for other highly variable glycoproteins such as hepatitis C virus envelope.

**Conclusions:**

NGlyAlign is an automated tool for mapping and building glycosylation motif libraries to accurately align highly variable regions in HIV sequences. It can provide the basis for many studies reliant on single robust alignments. NGlyAlign has been developed as an open-source tool and is freely available at https://github.com/UNSW-Mathematical-Biology/NGlyAlign_v1.0 .

## Background

Generating an automated and functionally optimal multiple sequence alignment (MSA) is a challenging task in HIV or in any sequence data analysis [1, 2]. The challenges of HIV-1 arise largely due to its remarkable ability to adopt high levels of mutation while maintaining fitness. The envelope glycoprotein may exhibit genetic variation of 15 to 20% within an individual subtype and 25 to 35% between subtypes [3]. Even within an individual, HIV continues to evolve resulting in the emergence of a quasi-species of virus. As a consequence of this variability, most of the alignment methods struggle to optimally align functional residues within the sequences, and therefore fail to summarize the overall properties of the protein domain. Alignments particularly fail in the hypervariable regions of the envelope gene, to the extent that these regions are often omitted when attempting to describe properties across individuals [4, 5]. Nevertheless it is these regions that portray modes of viral evolution in response to immune pressure via incorporation of insertions or deletions (indels) and N-linked glycosylation [6]. Improved methods are required to properly align these highly variable sequences to capture information on how they have functionally evolved or diverged due to immune pressure.

When determining relationships between sequences, the foundation is usually a phylogenetic tree based on a number of bootstrapped alignments of sequences, where mutational dynamics provide a basis for their evolution and relatedness. By itself this approach can lead to considerable uncertainty for investigations of the highly variable regions of HIV envelope. These regions are so variable that alignments are highly variable themselves and fail to provide a robust foundation for some analyses. Of particular concern are those investigations where a single alignment is required, rather than a statistical relationship between sequences as reflected in a bootstrapped phylogenetic tree. For example, protein structure studies using covariation of observed residues at different positions among a set of aligned sequences require a single alignment upon which to base their calculations [7–9]. In these analyses, it is not the larger relationship between sequences that is of most interest, but rather the functional information that is buried within the sequences, provided the alignment is sufficiently accurate. A single robust MSA is the basis for these analyses. Current methods fail to produce an accurate alignment of the variable regions in HIV.

In a functionally correct alignment, residues with the same function should be aligned optimally, irrespective of their similarities from convergent evolution [10]. Functionally related sites often involve more than a single residue, therefore as well as aligning at individual positions, a library of functional blocks is also required to ensure the MSA produces a reasonable final alignment. Of particular relevance for HIV envelope, N-linked glycosylation is essential for correct folding, structural rearrangement of gp120 and is correlated with immunological functions such as shielding virus from neutralizing antibodies. Glycans comprise a stretch of 3 amino acids with a specific structure [*NXT*/*S*], where *X* represents any amino acid except proline. Some of these glycans are conserved due to their functional importance, while others are highly variable, representing genetic diversity and evolution away from neutralizing antibodies against envelope glycoproteins [11]. Our approach seeks to utilize these glycosylation sites as functional constraints in alignment of the problematic variable regions.

A common and accepted practice in MSA is to manually enforce additional structural and functional information through expert domain knowledge. Considerable effort must be expended in manual editing these alignments, which becomes impractical for even moderate numbers of sequences when variability is high. Our central motivation is to design an automated approach that would necessitate no or limited manual editing. The approach developed here, builds an automated library of functional constraints by organizing similar glycosylation sites as block constraints and statistically conserved global sites as single site constraints. This library automatically enforces partial columns in a consistency-based method such as Dialign [12] and the need for editing of the MSA is substantially reduced.

We describe the method (NGlyAlign) and compare its performance using two HIV-1 envelope datasets, one containing gp160 sequences from both the founding infection and from an equivalent number of sequences from chronically infected individuals, and the other a reference set of HIV gp120 sequences. Compared to a number of MSA methods, our method showed better alignment of variable regions and less misalignment, that can be instrumental for coevolution and other studies.

### Implementation

#### Algorithm

The non-variable regions of Env are sufficiently conserved that most MSA perform to a similar standard. Where they differ is in the almost arbitrary alignment in the five variable regions. Addressing this problem is our main aim. Hence we assume a suitable MSA, such as HMMER [13, 14] or PSI-Coffee [10], has been used to align the Env sequences with the assumption that the alignment of the conserved regions is correct but the variable regions need additional adjustment. We then extract the separate variable regions from this preliminary MSA.

**Fig. 1:**
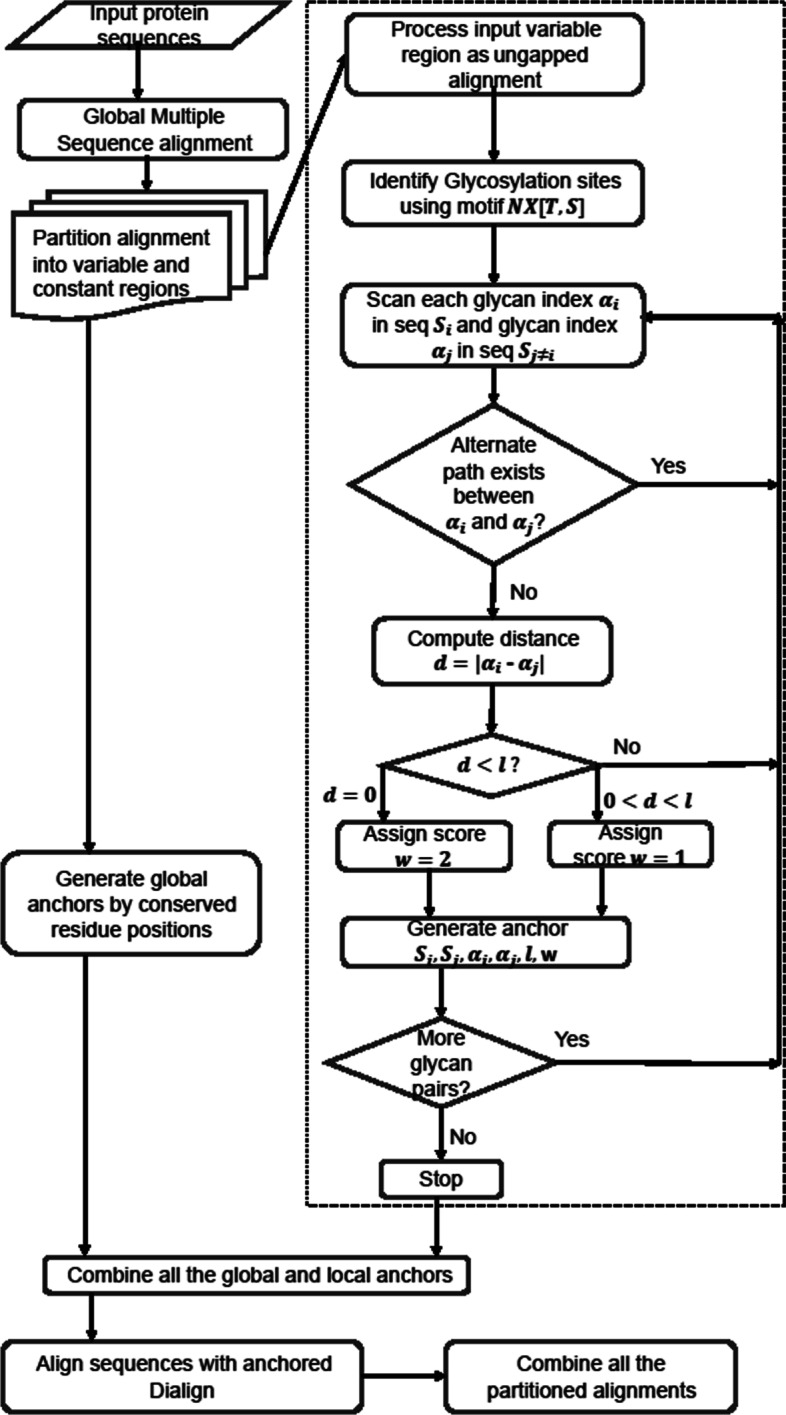
Flowchart of HIV-1 multiple sequence alignment. The NGlyAlign steps are shown within the dotted box

NGlyAlign accepts the sequences restricted to each variable region, removes the gaps inserted by the global alignment and then realigns them with the N-linked glycan constraints extracted as part of the library building procedure. To identify structural constraints, the percentage of identical residues in each global MSA column is computed. Columns with residue conservation score ≥ 99% are automatically selected as constraints. This conservation threshold can be varied to ≥95% as an option. The percentage identity is especially applicable when the sequence similarities are unknown (or highly divergent) and BLOSUM scoring cannot be applied. As an additional function, the deep scoring matrix BLOSUM50 is included and, based on the entropy in each MSA column, a structurally conserved site with entropy less than 0.85 can be selected as a constraint. An option to vary this entropy threshold is incorporated as a function for expert users. The method operates in the following three phases (Fig. **1**). In the first phase NGlyAlign identifies each N-linked glycosylation site in the input sequence set. In the second phase, a mathematical model is applied to extract a minimal equivalent set of block constraints. In the last phase, the conservation score is computed from the global alignment and all the constraints are assigned with appropriate priority scores to build the final library for consistency based MSA.

**Fig. 2:**
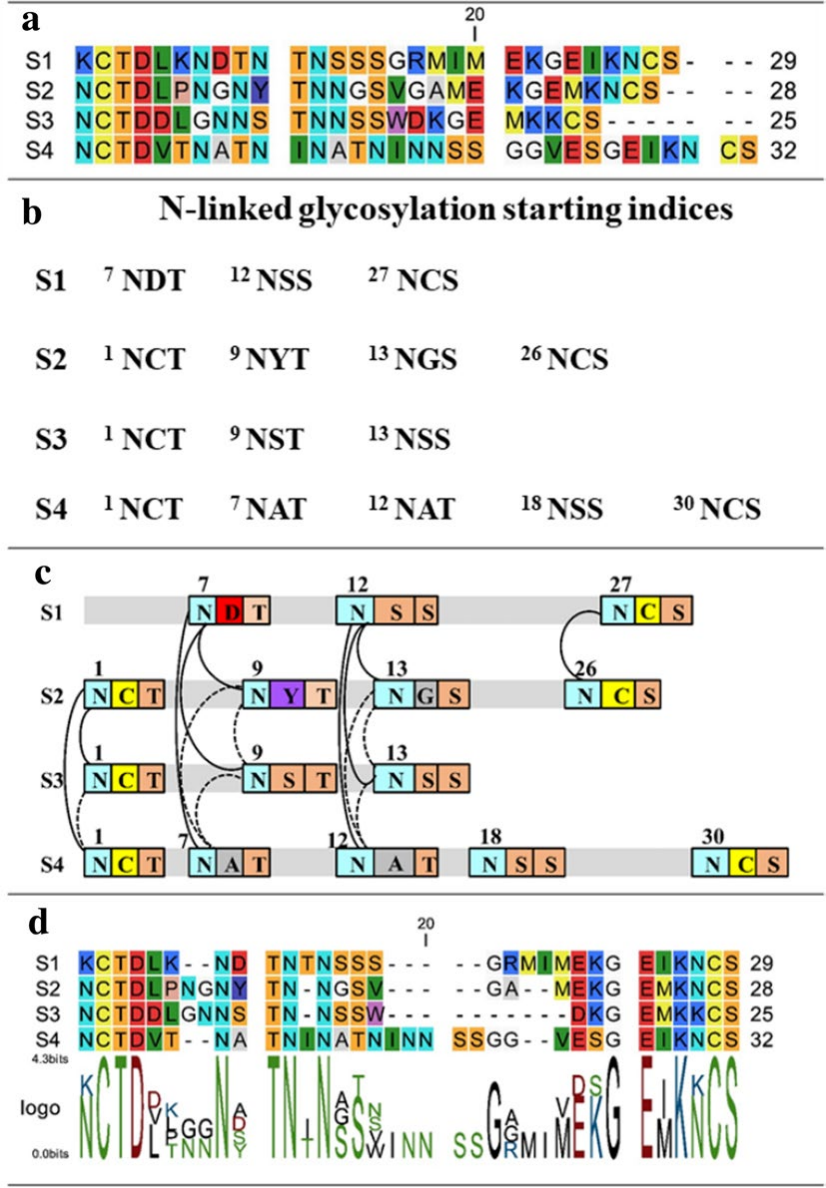
**a** Variable region sequences with gaps removed. **b** Table of glycosylation sites. **c** Candidate glycosylation anchors (solid and dashed lines). The dashed lines are removed through the transitive reduction step creating a smaller but equivalent set of anchors. **d** Final alignment resulting from using Dialign with these anchors

NGlyAlign identifies each N-linked glycan [*NXT*/*S*], where *X* can be any amino acid other than proline [15] (Fig. **2**a). In the case of two continuous glycosylation sites (e.g. NNST), only the second asparagine is marked as glycosylated. Each sequence is then represented as an ordered set of glycan positions (Fig. **2**b). Once the indexing is completed, a pair-wise sequence comparison is carried out over these indices using the reference sequence (HXB2 for HIV-1, GenBank accession number K03455) as a template and a set of glycosylation blocks is generated. A block constraint (*s*_1_, *s*_2_, α_1_, α_2_, *l*, *w*) can be described as an equal length (*l* = 3) segment over a pair of sequences *s*_1_, *s*_2_ with corresponding glycosylation starting positions α_1_ and α_2_, and priority score *w* (Fig. 2c).

The program successively searches glycan blocks within distance *d* in a pair-wise manner and blocks appearing at identical starting positions (∣α_1_ − α_2_ ∣ = *d* = 0) are assigned with priority score *w*_2_ otherwise, *w*_1_(<*w*_2_) for 0 < *d* < *l*. To avoid introducing too many gaps, the default search radius *d* is set with respect to the glycosylation motif length (*d* = *l* − 1). The resulting set of all glycosylation constraints can be viewed as a network of vertices, described by all the positions connected by block constraints, as well as the edges formed by the constraints (all solid and dashed lines in Fig. 2c). During the constraint selection process, transitive reduction [16] produces a forest within the network, containing edges (*u*, *v*) for which there is no alternate path from *u* to *v* and produces a minimal equivalent set of constraints (solid lines only in Fig. 2c).

To avoid inconsistent alignments in the gene boundaries, structurally known constraints, such as the positions which are ~ 99% conserved in the initial global alignment, are combined with a high priority score.

Generating a correct and small set of constraints as achieved in the transitive reduction step is important to form partial alignment columns for the final MSA. For a given set of library constraints and input sequences, Dialign greedily accepts a subset of constraints based on their weights and rejects inconsistent ones. Thus, the final alignment (Fig. **2**d) depends on how these weights are defined. The priority score weights in these calculations were conducted with *w*_1_ = 1, and *w*_2_ = 2. The structural constraints associated with conserved positions were also assigned a score of 2. Our representation provides glycosylation blocks as a chain of short conserved regions with priority weights which automatically form partial columns to align the glycosylation sites while the structural constraints maintain the boundary of the variable regions. NGlyAlign is implemented in MATLAB and the code and validation set are provided in Additional file 1.

### Data test sets

In total 156 (founder and chronic 78 each) HIV-1 type subtype B [17, 18], gp160 DNA sequences, converted to amino acid (AA) sequences (nt2aa, Matlab 2012b, The MathWorks Inc., Natick MA, USA) were combined for this work. The set was initially aligned through HMMER [13] using HIVAlign [14] against the HXB2 (GenBank accession number K03455) reference profile. Performance was validated using MEGAX [19] with global alignment tools ClustalW [20], Muscle [21], Clustal Omega [22] and T-Coffee [23].

A second test set consisted of 91 HIV-1 subtype B sequences from the first variable region of gp120 with diverse levels of cross-reactive neutralization activity [24]. This benchmark set had previously been validated with Praline [25] proving superior over other multi-alignment tools ClustalW [20] and Muscle [21]. However, the final alignment required extensive manual editing and we compare the ability of the NGlyAlign anchored method to reconstruct this benchmark alignment against Praline, HMMER using HIVAlign, Dialign2, Clustal Omega and T-Coffee.

### Methods of comparison

We applied the following widely used standard scores to measure quality of the alignment [26–28].

#### Sum of pairs score (SPS)

This score measures the number of residue pairs correctly aligned in the reference alignment. Let *T* be a test alignment of *N* sequences with *M* columns, *T*_*ij*_, 1 ≤ *i* ≤ *N*, 1 ≤ *j* ≤ *M*, being compared to a reference alignment *R* with residues *R*_*ij*_, 1 ≤ *i* ≤ *N*, 1 ≤ *j* ≤ *M*_*R*_. For the *j* th column, if a pair of nongap residues *T*_*ij*_ and *T*_*kj*_ are in the same column of the reference alignment *R*, then the score *P*_*jik*_ = 1, otherwise, *P*_*jik*_ = 0. The score *S*_*j*_(*T*, *R*) for the *j* th column in *T* is

$${S}_j\left(T,R\right)={\sum}_{i=1,i\ne k}^N\ {\sum}_{k=1}^N\ {P}_{jik}$$

The SPS for the full alignment with respect to the reference alignment with the *j*′ th column score *S*_*Rj*′_(*R*, *R*) (=*x*(*x* − 1)/2 where *x* is the number of nongap residues in the column) is

$$SPS\left(T,R\right)={\sum}_{j=1}^M{S}_j\left(T,R\right) \!\left/ {\sum}_{j\prime =1}^{M_R}\ {S}_{Rj\prime}\left(R,R\right)\right.$$

#### The modeler score (PS)

This is the reverse sum of pairs score and computed as the total number of residue pairs correctly aligned in *T* with respect to the reference alignment *R* divided by the total number of aligned residues in the alignment *T*

$$PS\left(T,R\right)={\sum}_{j=1}^M{S}_j\left(T,R\right)\!\left/ \!{\sum}_{j=1}^M\ {S}_j\left(T,T\right)\right.$$

#### Column score (CS)

This score is the percentage of columns where both alignments agree completely. For the *j* th column in the test alignment *T*, the column score *CS*_*j*_(*T*, *R*) = 1, if all the residues are matched to the reference alignment *R*, otherwise, *CS*_*j*_(*T*, *R*) = 0. The total column score for the full alignment

$$CS\left(T,R\right)={\sum}_{j=1}^{\left|T\right|}{CS}_j\left(T,R\right)\!\left/ \!\mid R\mid.\right.$$

#### Sum of entropy score as a measure of variability

Shannon’s information theoretic entropy is a widely used measure to assess MSA column quality [29–31]. Entropy of a column is computed as

$$S\left({m}_i\right)=-{\sum}_a{p}_{ia}{\log}_2{p}_{ia}$$

where *m*_*i*_ denotes the *i* th column of an alignment *m* and *p*_*ia*_ is the probability the residue *a* appears in column *i*. Maximal entropy occurs when all residue types are represented evenly whereas zero entropy indicates invariant columns. A better alignment will tend to match residues at each position and so will minimise the sum of entropy scores over all the columns.

### Computation

Each program was tested with its default settings. The AlignStat R package (http://alignstat.science.latrobe.edu.au//) and Jalview [32] were used as validation tools and for graphical displays. All programs were run on an Intel(R) Core(TM) i7–4790 CPU @ 3.60GHz, 4 Core(s) with Windows 10 Enterprise.

## Results

### Alignment comparisons of founder and chronic HIV-1B envelope

A wide selection of global alignment methods Muscle [21], ClustalW [20], ClustalOmega [22], HIVAlign [14] and T-Coffee [23] were executed with their default settings for a test set of 156 subtype B HIV-1 gp160 sequences, obtained from individuals at both the founder and chronic stages of infection. There is no ‘gold standard’ that we can use for this comparison as true alignments are unknown and no straight-forward measure to assess the quality of the alignment. Therefore, several methods of comparison were investigated. Firstly, accuracy within structurally conserved regions [33], especially against the 31 conserved glycosylation sites in HXB2 was compared across the methods. For each site *i*, conservation was computed as the number of times the glycosylation was observed (*G*_*i*_) *100 / total number of sequences (*N*). Given the focus on glycosylations sites, our method performed as well or better in all glycosylation sites except for four positions and was the unique maximum at four other positions (Table 1). The combined score over all the glycosylation sites exceeded the next best method HIVAlign by 9%. Clustal Omega performed the worst with around a 15% drop in conservation score compared to our method. Alignment characteristics such as percentage gaps, are listed in Supplementary Table 1A.

**Table 1:** Known HXB2 N-linked glycosylation site conservation by different alignment methods in HIV-1 protein B sequences (Bold value denotes equally maximum score and ^a^ when unique for our method)

HXB2 position	Location	NGlyAlign + Dialign	HIVAlign	ClustalW	Muscle	Clustal Omega	T-coffee
88 NVT	C1	**100**	**100**	98.72	**100**	**100**	**100**
136 NDT	V1	44.23	23.08	15.38	**52.56**	23.08	23.08
141 NSS	V1	**55.13**^**a**^	17.95	26.28	7.05	6.41	20.51
156 NCS	V1	**97.44**	**97.44**	**97.44**	**97.44**	**97.44**	**97.44**
160 NIS	V2	**89.1**	**89.1**	**89.1**	**89.1**	**89.1**	**89.1**
186 NDT	V2	**28.85**^**a**^	15.38	12.82	15.38	13.46	14.74
197 NTS	V2	**98.08**	**98.08**	**98.08**	**98.08**	**98.08**	**98.08**
230 NKT	C2	**26.28**	**26.28**	**26.28**	**26.28**	**26.28**	**26.28**
234 NGT	C2	**74.36**	**74.36**	**74.36**	**74.36**	**74.36**	**74.36**
241 NVS	C2	**97.44**	**97.44**	**97.44**	**97.44**	**97.44**	**97.44**
262 NGS	C2	**100**	**100**	**100**	**100**	**100**	**100**
276 NFT	C2	**98.08**	**98.08**	**98.08**	**98.08**	**98.08**	**98.08**
289 NTS	C2	**69.23**	**69.23**	**69.23**	**69.23**	**69.23**	**69.23**
295 NCT	C2	**78.85**	**78.85**	**78.85**	**78.85**	**78.85**	**78.85**
301 NNT	V3	**92.95**	**92.95**	**92.95**	**92.95**	**92.95**	**92.95**
332 NIS	C3	**87.18**	**87.18**	**87.18**	**87.18**	**87.18**	**87.18**
339 NNT	C3	77.56	77.56	77.56	77.56	77.56	**78.21**
356 NKT	C3	83.33	83.33	12.82	10.9	45.51	**87.18**
386 NST	V4	**82.05**	**82.05**	**82.05**	**82.05**	**82.05**	**82.05**
392 NST	V4	**87.82**	**87.82**	**87.82**	**87.82**	**87.82**	**87.82**
397 NST	V4	**89.10**^**a**^	8.97	39.1	37.18	8.97	8.97
406 NNT	V4	32.05	20.51	17.31	**50**	8.97	16.03
448 NIT	C4	**92.31**	**92.31**	**92.31**	**92.31**	**92.31**	**92.31**
463 NES	V5	**69.87**^**a**^	46.79	32.05	26.28	39.74	42.95
611 NAS	gp41	**98.08**	**98.08**	**98.08**	**98.08**	**98.08**	**98.08**
616 NKS	gp41	**89.74**	**89.74**	**89.74**	**89.74**	86.54	**89.74**
624 NHT	gp41	**95.51**	**95.51**	**95.51**	94.87	0.64	0.64
637 NYT	gp41	**98.72**	**98.72**	**98.72**	**98.72**	98.08	**98.72**
674 NIT	gp41	**13.46**	**13.46**	**13.46**	**13.46**	**13.46**	**13.46**
750 NGT	gp41	**1.28**	**1.28**	**1.28**	**1.28**	**1.28**	**1.28**
816 NAT	gp41	**69.87**	**69.87**	**69.87**	**69.87**	**69.87**	**69.87**
Combined score over N Sites		**2317.95**^**a**^	2131.4	2069.87	2114.1	1962.82	2034.62

**Fig. 3:**
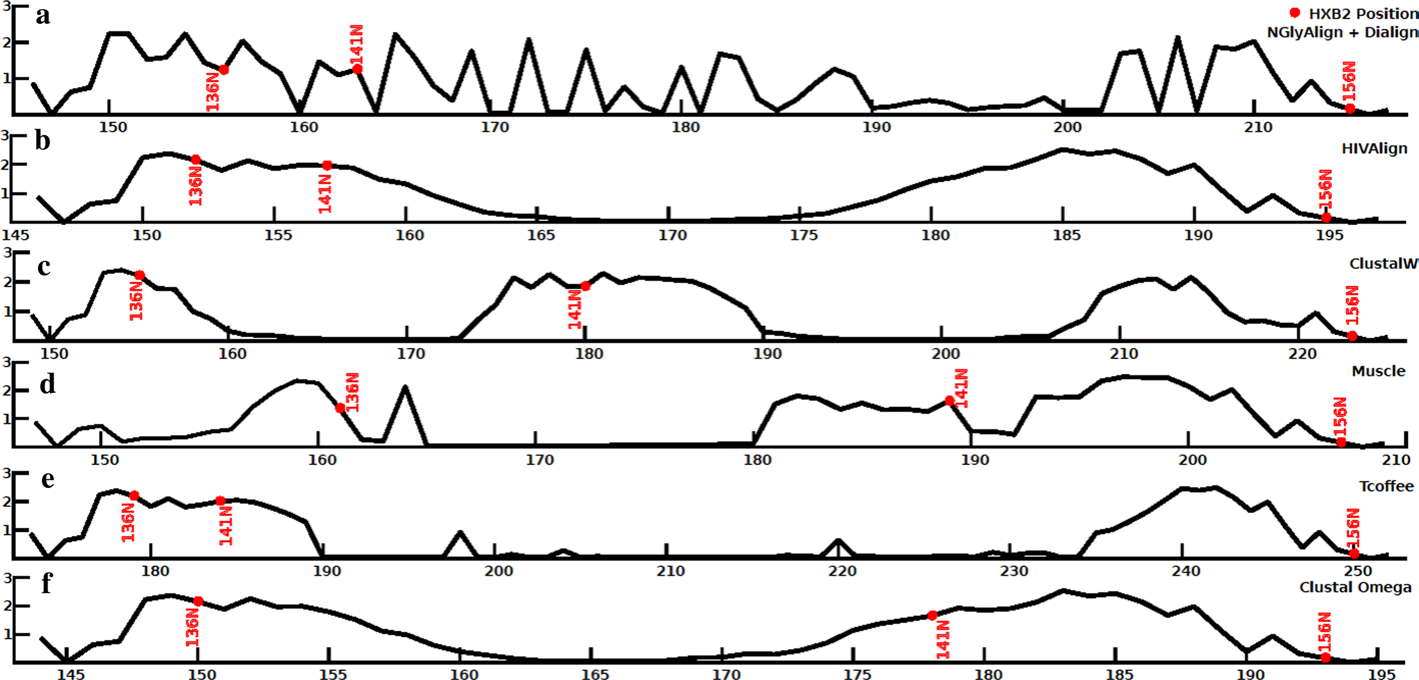
A comparison of entropy profiles for each method applied to the variable V1 region (HXB2 131–157) of 156 HIV-1 subtype B sequences. **a** NGlyAlign + Dialign (70 residues), **b** HIVAlign (50 residues), **c** ClustalW (75 residues), **d** Muscle (61 residues), **e** T-Coffee (78 residues), **f** Clustal Omega (50 residues). Note: for each plot, x-axis values are corresponding alignment positions by the method and y axis values are entropies at those positions. For comparison, the N-glycans at HXB2 positions 136, 141 and 156 are included in red

The alignments were also compared using the sum of total entropy scores over the 856 positions in the reference sequence HXB2. NGlyAlign achieved the minimum score 353.67, followed by Muscle 360.54, HIVAlign 367.03, T-Coffee 367.39, ClustalOmega 370.93 and ClustalW 375.44. A comparison of entropy profiles for the variable V1 region is shown in Fig. 3. We note the smooth transitions from zero to high entropy values produced by other methods, suggesting an almost random grouping of residues. In the absence of the anchors, glycosylation sites are scattered throughout the length of the variable regions (Supplementary Figure 1).

### Alignment comparisons of reference HIV-1B envelope V1

We also examined the accuracy of our method using a widely studied HIV-1 alignment consisting of 91 subtype B gp120 sequences with response to neutralization antibodies in the variable regions. This second task examined how closely the NGlyAlign anchored method could reconstruct the reference alignment without manual processing and the quality of the final alignment was compared across five standard multi-alignment programs: Praline, Dialign (without anchor), HivAlign, Clustal Omega, and T-Coffee.

NGlyAlign processed the input set and generated 426 block anchors from 396 N-linked glycosylation sites. Structurally conserved anchors were automatically identified from the global alignment by HivAlign for six fully conserved positions (C131, E153, and K155-S158). Altogether, 972 anchors were used for the final alignment.

For each multi-alignment tool, parameters such as alignment length, percentage of gaps and conservation score (Supplementary Table 1B) were compared and our method achieved the closest match with the benchmark alignment in terms of all these parameters. Praline, HIVAlign and T-Coffee resulted in 2.3% (absolute), 2.8 and 1.7% higher conservation scores with respect to the reference alignment by compressing gaps 11.1, 13.9 and 8.5% respectively. Unanchored Dialign and Clustal Omega decreased conservation scores (by 2.9 and 1.1% resp.) with 7.2% higher and a 3.9% lower gaps. Our method reconstructed the reference alignment best with slightly decreased (0.3%) conservation score and with minimally increased 1.8% gaps.

**Fig. 4:**
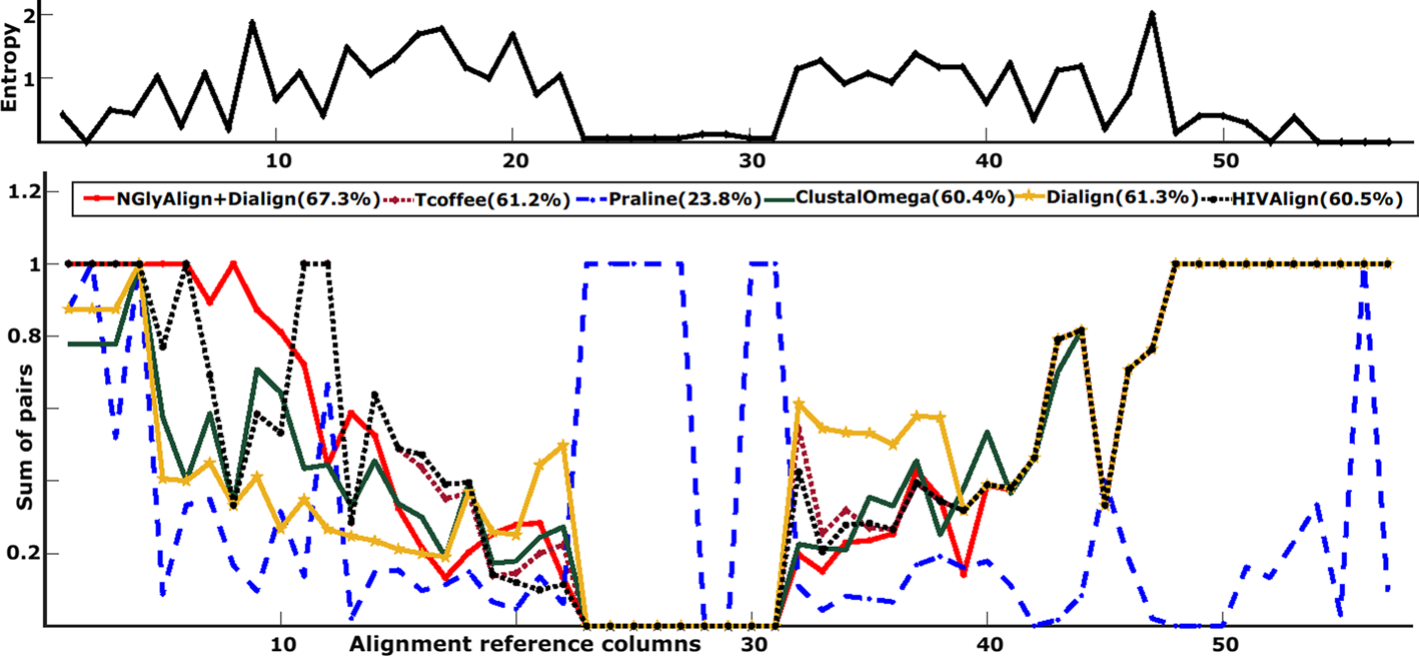
The reference alignment entropy for variable region 1 and the Modeler scores for each method

NGlyAlign anchored Dialign achieved the highest SPS score of 71.0% which is 44.3% higher than Praline, 4.2% higher than Dialign (no anchor), 1.8% higher than HIVAlign, 6.3% higher than Clustal Omega and 1.4% higher than T-Coffee (Supplementary Figure 2). Modeler score comparison (Fig. 4) also resulted in a maximal value for NGlyAlign being 43.5% higher in absolute terms than Praline, 6.9% higher than Clustal Omega, 6.8% higher than HIVAlign, 6.1% higher than T-Coffee, and 6.0% higher than Dialign (no anchor). Total CS score for our method was 23.7%, Praline 8.5%, Dialign (no anchor) 30.3%, HIVAlign 22.2%, Clustal Omega 22.6% and T-Coffee 24.5%. CS score by the AlignStat tool does not account for gaps and therefore, Dialign (no anchor) gained the highest CS score regardless of it producing the longest alignment with a high percentage of gaps. NGlyAlign consistently reconstructed the reference alignment within the highly variable regions with reasonable SPS, Modeler and CS scores (for details see Supplementary Figure 2). The aligned sequences with high-lighted glycans are show in Supplementary Figures 3 to 9.

NGlyAlign uses glycosylation and conserved blocks as anchors in the alignment process so its performance may be more susceptible to deletions. To investigate this, the V1 region in 28 of these sequences were either subjected to single AA deletions in each sequence, or the first 3 AA of 7 of the sequences were deleted (all glycosylation blocks, Supplementary Figure 10), In comparison to HIVAlign, the NGlyAlign alignments still were closest to reproducing the alignment scores of the original reference alignment (Supplementary Table 2). Although deletions will impact the alignment quality of NGlyAlign, these will adversely affect other methods as well.

### Estimating performance

**Fig. 5:**
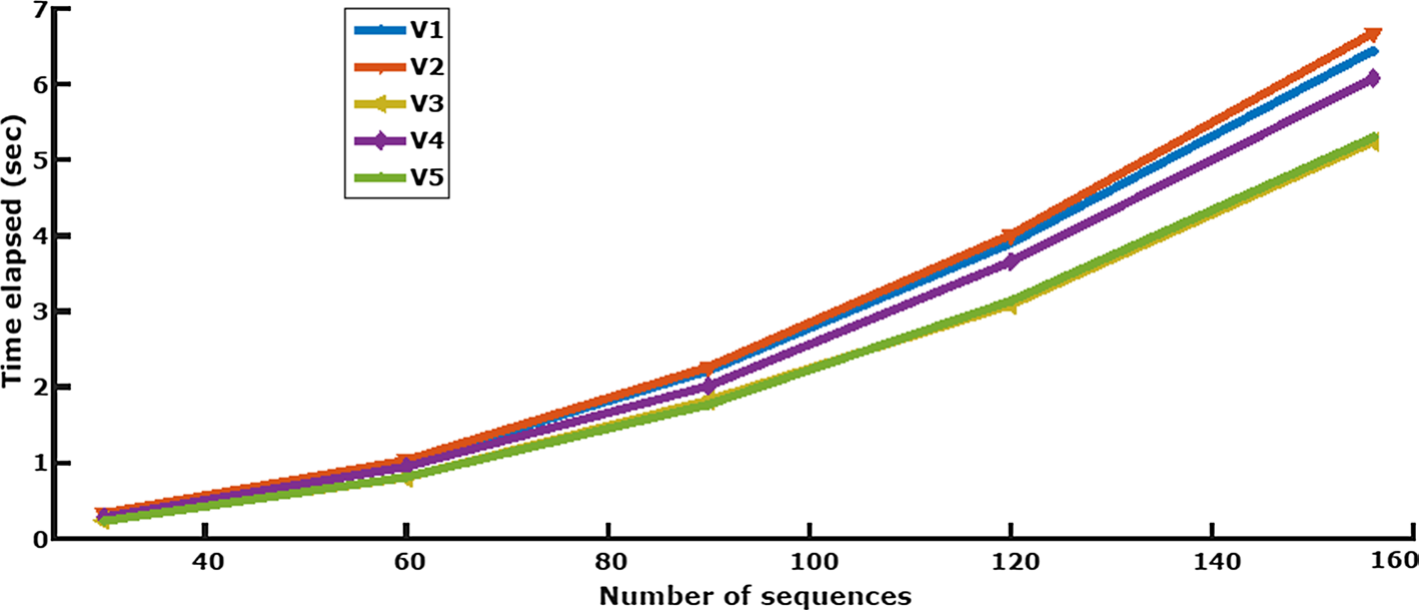
Execution time for NGlyAlign anchors in gp120 variable regions

We analysed NGlyAlign’s performance for different numbers of sequences in all five variable regions, determining the execution time required for glycan anchor generation. Our method aligns similar glycosylation sites in a pair-wise manner, so computation time increases quadratically with the number of sequences. Running time also grew approximately linearly with the maximum sequence length in each variable region (Fig. **5**). NGlyAlign added an average 13.14% overhead on top of Dialign’s computation time (Supplementary Table 3).

## Discussion

Two different HIV datasets were aligned with NGlyAlign and compared to other state-of-the-art methods. The results showed that pre-alignment of glycosylation blocks with NGlyAlign generally performed better in aligning highly variable HIV regions. Our methods achieved superior performance on a number of measures including the lowest entropy over the 156 gp160 sequences, and being closest to reproducing the reference gp120 alignment, but without the additional requirement of manual editing of the initial MSA. Although NGlyAlign adds an overhead in that it requires an initial MSA followed by NGlyAlign’s separate alignment of the variable regions, the improvement in the alignment more than compensates for the small additional computation (Fig. **5**).

The need to improve alignments for these highly variable sequences is important for analyses that require a single alignment rather than determining relationships between sequences through a bootstrapped phylogenetic tree. Examples of such analysis include determining the fitness landscape of the HIV envelope protein, and in assessing the compensatory mutational changes of HIV Env as immune pressure forces the protein away from vulnerable structures early in transmission. Each of these cases require considerable computation but must base these computations on a single alignment. The regions that are the most variable are also the most prominent in reflecting these changes whether they be concerned with fitness or immune escape. From the almost random placement of residues in these regions (Fig. 3), no amount of bootstrapping will produce a robust foundation for these types of investigations. Furthermore, the motifs that are fundamental in altering the envelope protein and its accessibility to antibodies are the glycans that are used in NGlyAlign to improve sequence alignments. This alignment with glycans also best-matched the reference alignment of HIV gp120 (Supp Table 1), and achieved this result without manual editing. Manual editing for reasonable numbers of sequences of the generally poorly aligned variable regions is prohibitive in time, and ultimately subjective in nature. Our method eliminates the need for manual editing and results in an objective alignment based on the biological mechanisms driving Env evolution.

Our quantitative benchmarking showed that variable and disordered HIV regions clearly benefit from NGlyAlign. Methods that aim for accurate phylogenetic reconstruction and global sequence similarity, even with homology profiling such as PSI-Coffee, Praline, HIVAlign are susceptible to over-alignment [34, 35] where deceptive higher similarity scores are achieved by re-aligning ambiguous residues. In addition, repetitive glycosylation motifs occurring in different number of copies per sequence makes the situation worse. Therefore, our method based on anchoring these disordered regions based on available conserved motifs had the advantage in constructing a functionally meaningful alignment within the consistency-based Dialign framework.

NGlyAlign represents glycosylation blocks as rigid motifs without indels. Due to the explosive number of possible gap variations, most motif discovery methods are limited to ungapped motifs with only variations in substitutions. In cases where gaps are allowed, motif discovery against known databases such as PROSITE [36] is carried out in the initial step, followed by a separate alignment method in a semi-automated manner. This is a computationally extensive task. Only a handful of methods attempt to discover variable motifs and with limited success [35, 37, 38].

Although this method has been developed to align HIV envelope sequences, it is applicable to other viral glycoproteins whose variability is associated with the presence of N-linked glycosylation sites. NGlyAlign can also be extended for other motifs by altering the input search pattern. The E1E2 envelope glycoprotein of hepatitis C virus (HCV) also exhibits highly variable regions and is heavily glycosylated [39]. As with HIV envelope, HCV envelope generates additional glycosylation sites in response to immune pressure [34]. Our method can produce alignments in both these cases that relevantly describe the regions responsible for vaccine and immune escape.

## Conclusions

NGlyAlign is an automated tool that builds glycosylation motif libraries and accurately aligns highly variable regions in HIV sequences by creating partial columns in consistency-based MSA methods such as Dialign. We propose that NGlyAlign can provide essential basis for many studies reliant on single robust alignments.

## Supplementary Information

**Additional file 1.** NGlyAlign tool in matlab along with a test file and manual is included as additional files.

## Data Availability

Project Name: NGlyAlign. Project Home Page: https://github.com/UNSW-Mathematical-Biology/NGlyAlign_v1.0 Programming Language: MATLAB. Other requirements: Matlab Runtime version 9.8 (R2020a) Dependency: Dialign2 (.exe is included with the app). License: Free for non-commercial purposes. Any restriction to use by non-academics: None.

## Abbreviations

HIV: Human Immunodeficiency Viruses
HCV: Hepatitis C Virus
MSA: Multiple Sequence Alignment
SPS: Sum of Pairs Score
CS: Column Score
SP: Sum-of Pairs
gp41: glycoprotein 41
