# Supplementary material for: NGlyAlign: an automated library building tool to align highly divergent HIV envelope sequences
Source: BMC Bioinformatics. 2021 Feb 8;22:54. doi: 10.1186/s12859-020-03901-y (PMC7869453; doi:10.1186/s12859-020-03901-y)
Supplement: Supplementary file 1 — Additional file 1. NGlyAlign tool in matlab along with a test file and manual is included as additional files. [file 12859_2020_3901_MOESM1_ESM.docx]

NGlyAlign: an automated library building tool to align highly divergent HIV envelope sequences

Elma H Akand^1^* and John M Murray^1^

^1^School of Mathematics and Statistics, UNSW Sydney, NSW 2052, Australia

Supplementary Material

Table 1 Alignment characteristics of different methods for A) Founder and Chronic HIV-1B gp160 sequences, and B) 91 HIV-1 B gp120 variants from 21 patients with diverse levels of cross-reactive neutralization activity

| **Dataset** | **Method** | **Alignment Length** | **% Gaps** | **% Conserved** | **% Columns ungapped** |
| --- | --- | --- | --- | --- | --- |
| (A) Founder and Chronic HIV-1B (Full length) | NGlyAlign + Dialign | 1078 | 20.5 | 17.4 | 62.1 |
|  | HIVAlign | 987 | 13.2 | 19.3 | 69.6 |
|  | ClustalW | 996 | 14 | 18.3 | 66.7 |
|  | Muscle | 989 | 13.4 | 18.9 | 69.7 |
|  | T-coffee | 1028 | 16.7 | 18.3 | 68.5 |
|  | Clustal Omega | 954 | 10.2 | 19.3 | 73.7 |
| (B) Cross-reactive neutralization HIV-1B (V1 region) | Reference Alignment | 57 | 47.7 | 10.5 | 21.1 |
|  | NGlyAlign + Dialign | 59 | 49.5 | 10.2 | 25.4 |
|  | Praline | 47 | 36.6 | 12.8 | 29.8 |
|  | Dialign (no anchor) | 66 | 54.9 | 7.6 | 10.6 |
|  | HIVAlign | 45 | 33.8 | 13.3 | 46.7 |
|  | T-coffee | 49 | 39.2 | 12.2 | 36.7 |
|  | Clustal Omega | 53 | 43.8 | 9.4 | 17 |

|  |
| --- |
|  |
|  |
|  |
|  |
|  |
|  |

Figure 1 Placement of HXB2 glycans in alignments of variable regions V1- V5 of gp120 from HIV-1 B 156 strains using methods: *a*) NGlyAlign anchored Dialign, *b*) HIVAlign, *c*) ClustalW, and *d*) Muscle *e*)ClustalOmega *f*)T-Coffee

| a)   |  |
| --- | --- |
| b)   |  |
| c)   |  |
| d)   |  |
| e)   |  |
| f)   |  |

Figure 2 Similarity heatmaps, Sum of pairs Scores(PS) and Column Scores(CS) for reference vs a) NGlyAlign anchored Dialign b) Praline c) Dialign (no anchor) d) HIVAlign e) Clustal Omega and f) T-coffee. In the heatmap, each column of the two alignments is shown. The dark diagonal line represents high consensus and parallel grey lines potential conflict regions*.*

|   Figure 3 Reference alignment with glycosylation sites highlighted as beige. Alignment length=57 residues, gaps=47.7%, conserved=10.5% and ungapped columns=21.1%. |   Figure 4 result from NGlyAlign with glycosylation blocks highlighted as beige. Alignment length=59 residues, gaps=49.5%, conserved=10.2% and ungapped columns=25.4%. |
| --- | --- |
|   Figure 5 result from Praline with glycosylation sites highlighted as beige. Alignment length=47 residues, gaps=36.6%, conserved=12.8% and ungapped columns=29.8%. |   Figure 6 result from Dialign (no anchor) with glycosylation sites highlighted as beige. Alignment length=66 residues, gaps=54.9%, conserved=7.6% and ungapped columns=10.6%. |
|   Figure 7 result from HivAlign with glycosylation sites highlighted as beige. Alignment length=45 residues, gaps=33.8%, conserved=13.3% and ungapped columns=46.7%. |   Figure 8 result from T-coffee with glycosylation sites highlighted as beige. Alignment length=49 residues, gaps=39.2%, conserved=12.2% and ungapped columns=36.7%. |
|   Figure 9: result from Clustal Omega with glycosylation sites highlighted as beige. Alignment length=53 residues, gaps=43.8%, conserved=9.4% and ungapped columns=17%. |  |


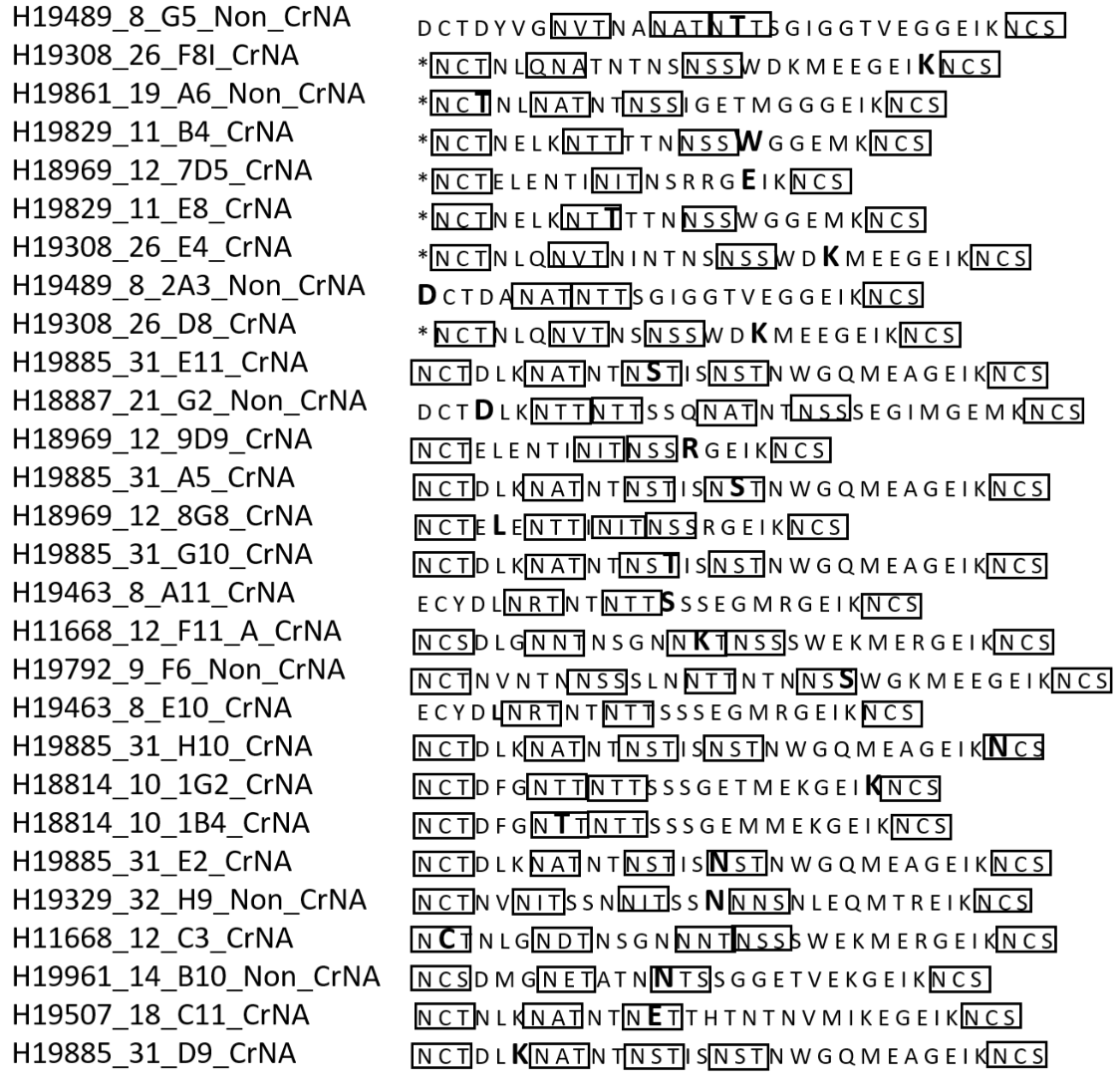


Figure 10: Deletion experiments and their effect on alignment were conducted on a set of 28 sequences with marked glycosylation sites in boxes. Randomly deleted single residues are in bold letters. For the block deletion investigation, the first 3AA of the 7 sequences (* marked) were deleted.

**Supplementary Table 2**: Results of alignment after single or block deletion using NGlyAlign or HIVAlign compared to the reference alignment of 28 V1 sequences.

|  | **Before deletion** | | | **After single deletion** | | **After block deletion** | |
| --- | --- | --- | --- | --- | --- | --- | --- |
| **Parameters** | **Reference** | **NGlyAlign** | **HIVAlign** | **NGlyAlign** | **HIVAlign** | **NGlyAlign** | **HIVAlign** |
| Length | 57 | 52 | 48 | 56 | 42 | 53 | 50 |
| % Gaps | 47.7 | 42.7 | 37.9 | 48.5 | 31.4 | 45.2 | 41.9 |
| % Conserved | 28.1 | 13.5 | 12.5 | 3.6 | 4.8 | 11.3 | 10.0 |
| % Ungapped | 22.8 | 28.8 | 33.3 | 10.7 | 11.9 | 20.8 | 20.0 |
| Entropy | 35.6 | 32.1 | 31.8 | 37.0 | 33.9 | 34.8 | 34.8 |

| **Supplementary Table 3:** execution time for NGlyAlign anchor generation with varying number of sequences in variable regions V1-V5. | | | | | | |
| --- | --- | --- | --- | --- | --- | --- |
|  | **Max. seq Length** | **No. of sequences** | **No. of glycans** | **No. of Anchors** | **NGlyAlign (sec)** | **Dialign (sec)** |
| ***V1*** | **52** | 30 | 123 | 134 | 0.30 | 1.00 |
|  |  | 60 | 251 | 301 | 1.01 | 5.00 |
|  |  | 90 | 389 | 490 | 2.22 | 11.00 |
|  |  | 120 | 520 | 668 | 3.90 | 22.00 |
|  |  | 156 | 665 | 858 | 6.45 | 46.00 |
| ***V2*** | **60** | 30 | 65 | 70 | 0.33 | 3.00 |
|  |  | 60 | 135 | 158 | 1.03 | 17.00 |
|  |  | 90 | 206 | 250 | 2.26 | 38.00 |
|  |  | 120 | 271 | 336 | 4.01 | 71.00 |
|  |  | 156 | 352 | 447 | 6.68 | 127.00 |
| ***V3*** | **40** | 30 | 50 | 48 | 0.23 | 3.00 |
|  |  | 60 | 105 | 103 | 0.80 | 14.00 |
|  |  | 90 | 158 | 156 | 1.82 | 33.00 |
|  |  | 120 | 209 | 207 | 3.09 | 60.00 |
|  |  | 156 | 274 | 272 | 5.24 | 111.00 |
| ***V4*** | **44** | 30 | 141 | 140 | 0.28 | 1.00 |
|  |  | 60 | 277 | 283 | 0.95 | 5.00 |
|  |  | 90 | 408 | 421 | 2.01 | 13.00 |
|  |  | 120 | 538 | 567 | 3.66 | 24.00 |
|  |  | 156 | 700 | 747 | 6.08 | 52.00 |
| ***V5*** | **23** | 30 | 43 | 48 | 0.23 | 0.90 |
|  |  | 60 | 80 | 100 | 0.81 | 2.00 |
|  |  | 90 | 127 | 165 | 1.77 | 6.00 |
|  |  | 120 | 167 | 221 | 3.14 | 12.00 |
|  |  | 156 | 217 | 289 | 5.30 | 24.00 |
